# Presentation, Diagnosis, and Treatment of a Sporadic Gastrinoma With Liver Metastases

**DOI:** 10.7759/cureus.61426

**Published:** 2024-05-31

**Authors:** Alisha Jadhav, Mumen Ayyat, Francesco M Serafini

**Affiliations:** 1 Surgery, St. George's University School of Medicine, Brooklyn, USA; 2 Surgery, Brookdale University Hospital Medical Center, Brooklyn, USA; 3 General Surgery, Brookdale University Hospital Medical Center, Brooklyn, USA

**Keywords:** distal pancreatectomy (dp), duodenal ulcers, zollinger-ellison syndrome, metastatic gastrinoma, gastrinoma

## Abstract

A gastrinoma is a rare and potentially deadly tumor. Gastrinomas can be difficult to detect at first, given that affected patients can present with nonspecific symptoms, such as anemia, weight loss, and diarrhea, leading to a large list of differentials. The symptoms can be hard to manage, and the treatment almost always includes surgical intervention. Gastrinomas often metastasize to the liver, in which case, the only curative treatment option is surgical resection of the primary tumor along with as many metastatic lesions as possible. This report reviews the case of a 59-year-old female who presented with symptoms of anemia and an upper gastrointestinal bleed, who was discovered to have a pancreatic gastrinoma with more than 12 liver metastases. It also describes the management of her critical condition, which was used to give her the best chance of survival.

## Introduction

A history of recurrent gastric or duodenal ulcers could indicate a gastrinoma, also known as Zollinger-Ellison syndrome (ZES). A gastrinoma is a neuroendocrine tumor that causes inappropriate gastrin secretion, ultimately causing gastric acid hypersecretion. This leads to symptoms of acid reflux, gastric and duodenal ulcers, and anemia. For a diagnosis of ZES to be confirmed, there must be evidence of hypersecretion of gastrin and a decreased gastric pH. A serum gastrin level of >1000 pg/mL and a gastric pH of 2 are diagnostic of gastrinoma. Specific studies, such as nasogastric tube fluid aspiration analysis or upper GI endoscopy, can be used to measure the pH of the stomach. A computed tomography (CT) scan, magnetic resonance imaging (MRI), or endoscopic ultrasound scans can then be used to localize the primary tumor. It should be noted that CT scans are not sensitive to small liver lesions, so MRI is a better option if metastatic disease is suspected. Another technique used to localize gastrinomas is the octreotide scan or somatostatin receptor scintigraphy. This involves the administration of radio-labeled octreotide, which has selective binding to somatostatin receptors found on gastrinoma tumor cells. It has high sensitivity and specificity for primary tumor detection and metastatic lesions. Combined with single-photon emission CT, this has shown an even higher sensitivity and specificity than CT or octreotide scan imaging alone [1,2].

Gastrinomas are located mostly in the gastrinoma triangle; the superior border of the triangle is the confluence of the cystic and common bile ducts, the inferior border is the second and third portions of the duodenum, and the medial border is the neck of the pancreas [2]. In most cases, pancreatic lesions have worse prognoses than duodenal lesions and are more often associated with metastatic disease. About 25-33% of patients who have gastrinomas develop them secondary to a syndrome called multiple endocrine neoplasia type 1 (MEN-1) syndrome [3]. This condition involves tumors of the parathyroid gland, pituitary gland, and pancreas. The management of sporadic gastrinomas compared to those associated with MEN-1 syndrome varies slightly, so it is important to distinguish between the two during the initial diagnosis. In patients with MEN-1, in addition to symptoms of gastrin hypersecretion, there would be signs of hyperparathyroidism, such as hypercalcemia, and symptoms of a pituitary adenoma. MEN-1 syndrome with hyperparathyroidism leads to increased serum calcium, a known stimulator of gastrin release from gastrinomas. Because of this, patients with MEN-1 syndrome have increased resistance to anti-secretory medical treatments and require different doses of treatment drugs than patients who have sporadic ZES. Another difference between MEN-1-related gastrinomas and sporadic gastrinomas is that the former is usually associated with duodenal lesions while the latter lesions usually localize in the pancreas. Sporadic pancreatic gastrinomas metastasize to regional lymph nodes in approximately 60% of patients and to the liver in 10-20% of patients, a much higher frequency than that of duodenal gastrinomas. The 10-year survival rate of those patients with sporadic disease is therefore much lower than in patients with duodenal gastrinoma [3,4].

Symptomatic treatment starts with proton pump inhibitors (PPIs), however, in cases where the gastrinomas metastasize, PPIs alone are inadequate for relief. Octreotide injections can also stabilize gastrin secretion symptoms, thereby reducing symptoms, but surgery is the only curative treatment. Surgery is useful in controlling the increased acid secretion by directly removing the secreting cells of gastrin and any metastases. If the metastases are unresectable, however, resecting the primary tumor is still beneficial as long as 80-90% of the mass can be safely removed [5]. After the procedure, it is important to monitor gastrin and chromogranin A, a neuroendocrine biomarker, levels to check for recurrence or incomplete removal of the disease. In this case report, we present a 59-year-old female with a history of recurrent duodenal ulcers only to be found to have a pancreatic gastrinoma with extensive liver metastasis. Gastrinomas with liver metastasis are associated with significantly reduced survival, therefore, our patient’s best chance of improving her survival rate was to undergo aggressive resection of the tumor and its associated liver metastases.

## Case presentation

**Figure 1:**
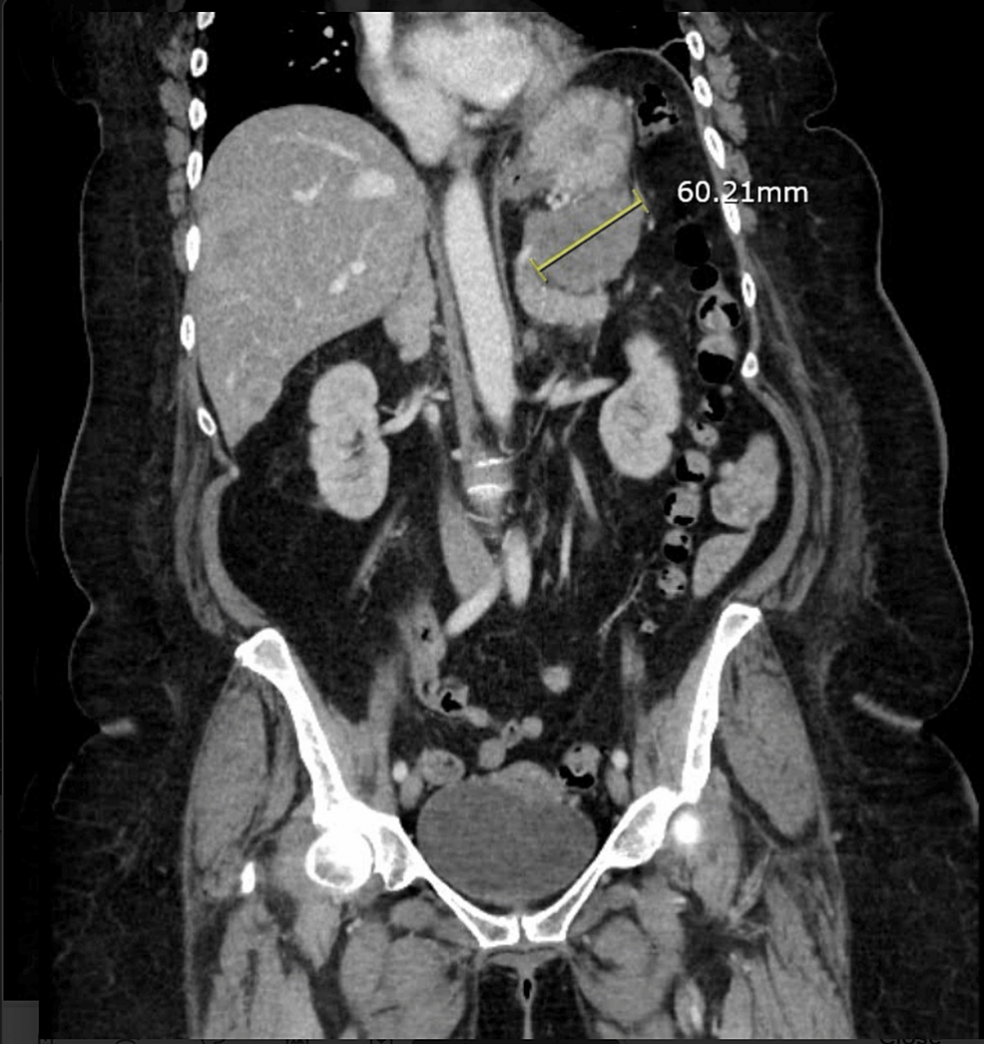
CT chest, abdomen, and pelvis frontal section displaying a 6.0 cm pancreatic tail mass with multiple liver metastases

**Figure 2:**
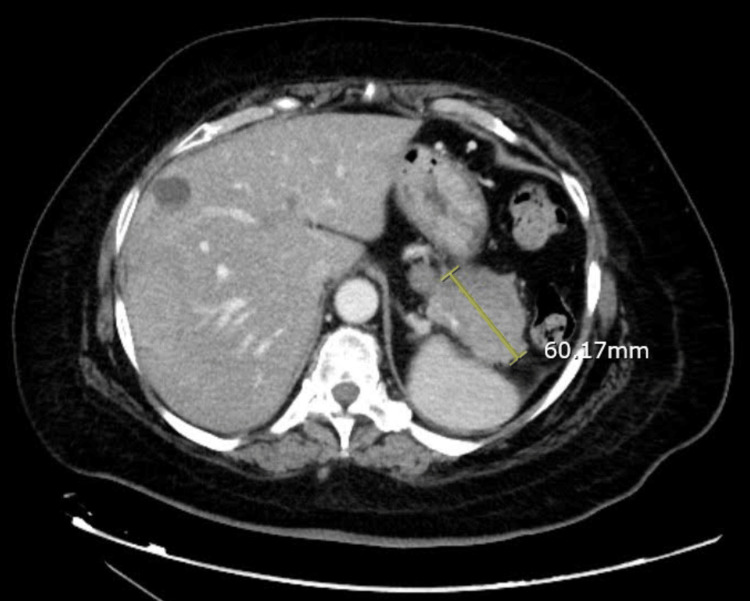
CT chest, abdomen, and pelvis transverse section displaying a 6.0 cm pancreatic tail mass with multiple liver metastases

A 59-year-old female with a past medical history of hypertension, gastroesophageal disease, and a perforated duodenal ulcer presented to the ED with epigastric pain, nausea, vomiting, and melena in November 2023. A CT scan of the chest, abdomen, and pelvis showed a 6 cm pancreatic tail mass with numerous liver lesions (Figure 1 and Figure 2). The patient continued to pass dark stools throughout her hospital stay. An esophagogastroduodenoscopy (EGD) done a few days later showed a non-bleeding 2 cm duodenal ulcer. The ulcer was treated with bipolar cautery. The second, third, and fourth portions of the duodenum were normal. Helicobacter pylori (H. pylori) testing came back negative. Her EGD done in 2022 also showed evidence of gastritis, esophagitis, and two ulcers in the duodenum, indicating a chronic history of peptic ulcer disease. The patient, however, continued to have symptomatic anemia and pass dark stools. A mesenteric angiogram with gastroduodenal artery embolization was done, and the patient noted relief of melena; she was discharged to follow up as an outpatient. Five days post-discharge, the patient came back to the ED presenting with weakness, inability to ambulate, and melena as of that morning. She was initially hypotensive, but this improved with fluids. Her carcinoembryonic antigen (CEA), carbohydrate antigen 19-9 (CA 19-9), and alpha-fetoprotein (AFP) were negative. Studies revealed gastrin levels to be 2745 pg/mL, raising concern for her pancreatic mass to be a gastrinoma. MRI showed a 5.0 mass in the tail of the pancreas, and multiple masses within the liver measuring up to 6.0 cm, confirming the diagnosis of a gastrinoma with liver metastases. She was then started on octreotide three times a day. The patient was scheduled for a distal pancreatectomy and liver metastasectomy. Platelets and hemoglobin remained stable, so the patient was taken for surgery. During the procedure, the abdomen was inspected and no diffuse carcinomatosis was found. The tumor at the distal pancreas was visualized. The pancreas was dissected off the retroperitoneum. The tumor was taken out, sent for a frozen section, and came back with negative margins. Twelve hepatic tumor metastases were resected. The procedure was complicated by bleeding from a previously diagnosed duodenal ulcer. The source of the bleeding was ligated after making a duodenotomy and suturing the bleeders using a three-point ligation technique.

Post-procedure, the patient's hospital course was complicated by a biochemical pancreatic fistula that self-resolved. She required a tracheostomy to get her off ventilation. She endorsed total relief of her previous symptoms of epigastric tenderness, nausea, vomiting, and melena. The patient was then decannulated and discharged to a rehab facility.

## Discussion

With our patient’s history of peptic ulcer disease, her symptoms when she initially presented to the Emergency Department, and her increased gastrin levels, the patient had a classic presentation of pancreatic gastrinoma. Imaging confirmed the diagnosis and the presence of liver metastasis, and after a multidisciplinary board discussion, the decision was made to proceed with surgical resection.

In cases that are associated with MEN-1 syndrome, diagnosis of the presence of a gastrinoma is not as straightforward as many patients with MEN-1 only show symptoms of ZES after the development of hyperparathyroidism. Hypercalcemia secondary to hyperparathyroidism can be the first sign of MEN-1, and it is directly associated with the later development of hypergastrinemia [6]. As it stands today, surgery is the only curative management for gastrinoma with liver metastasis. PPIs and H2 blockers are considered for symptomatic management during the early stages of the disease, when the gastrinoma and/or metastases are not resectable, or in cases where there is widespread metastasis.

Hepatic and/or gastroduodenal artery embolization is another therapeutic option to reduce metastatic symptoms as well as control peptic ulcer symptoms. Liver metastases get their blood supply from the hepatic artery so embolization of the hepatic artery can reduce symptoms of the metastases. One must factor in the possibility of postembolization syndrome, however, which can present as fever, nausea, loss of appetite, and abdominal pain. However, in cases as severe as this one, such a risk does not outweigh the benefits of the procedure [7].

As true with most metastatic diseases, the treatment options are limited. Chemotherapy is an option for patients with distant metastases; however, this treatment method has limited effects on disease regression and can prove to be more toxic than helpful to the patient. Studies also show that octreotide administration can control the growth of liver metastases and stabilize serum gastrin secretion, but this will not rid the patient of the disease itself [8]. Cingam et al. (2023) state that targeted therapies such as antiangiogenic modalities, multi-kinase, or mTOR inhibition are upcoming new approaches to tackling delayed tumor progression in patients with metastatic disease [9]. These novel therapies are still in the trial phase, however, so the most effective treatment for gastrinoma is still surgical resection.

Lastly, orthotopic liver transplantation is currently being explored as an option for patients who have limited liver metastatic resectibility. The survival rate is similar to that of hepatic resection, however, this needs to be studied further before it can be a definitive option for such patients [10].

The extent and location of metastases are the most important determining factors for mortality. For metastatic disease, removal of the primary tumor and as many metastases as possible is the goal to prevent further spread of the disease and improve survival. As our patient did have extensive liver metastasis, there is a possibility that the disease has not been completely eradicated. Good postoperative care and consistent follow-up are key for this patient; any sign of lingering disease or recurrence should be caught early and treated immediately. Checking serum gastrin levels and chromogranin A regularly post-surgery is vital to monitor the disease regression.

## Conclusions

Recognizing that a patient has ZES can be a challenge within itself. It can take a long time to diagnose, as the symptoms initially appear as non-specific, manifesting as abdominal pain, chronic diarrhea, gastroesophageal reflux disease (GERD), and anemia. In patients with chronic or recurrent ulcers, the diagnosis of gastrinoma should be high on the list of differentials. If applicable, symptomatic management with PPIs and imaging would be the next steps to identify the tumor and the extent of its metastases. Surgical resection remains the only curative treatment; however, with upcoming advances in medicine, it may become possible to rely more on medical treatments to aid in the curative process of ZES with liver metastases.
